# Atezolizumab‐induced fulminant type 1 diabetes mellitus occurring four months after treatment cessation

**DOI:** 10.1002/rcr2.685

**Published:** 2020-11-15

**Authors:** Toshihiko Nishioki, Motoyasu Kato, Shunichi Kataoka, Keita Miura, Tetsutaro Nagaoka, Kazuhisa Takahashi

**Affiliations:** ^1^ Department of Respiratory Medicine Juntendo University Graduate School of Medicine Tokyo Japan

**Keywords:** Atezolizumab, fulminant type 1 diabetes mellitus, immune‐related adverse events, lung adenocarcinoma

## Abstract

Atezolizumab is an immune checkpoint inhibitor (ICI) that is often associated with the development of several immune‐related adverse events, including fulminant type 1 diabetes mellitus (F1DM). Here, we present the case of a 73‐year‐old woman who was diagnosed with lung adenocarcinoma after surgical lung lobectomy. Two years later, she developed pulmonary metastasis, and atezolizumab treatment was initiated after seven years. However, she only completed two cycles of atezolizumab treatment because of disease progression. Four months after the interruption of atezolizumab treatment, she presented to the emergency department with fatigue and vomiting. On admission, she had a serum glucose level of 962 mg/dL, metabolic acidosis, and elevated ketone body levels. She was diagnosed with diabetic ketoacidosis induced by atezolizumab treatment. Her symptoms improved by insulin therapy. When ICIs are administered, care should be taken regarding the development of F1DM.

## Graphical abstract

We present a case of atezolizumab‐induced fulminant type 1 diabetes mellitus (F1DM) that occurred four months after withdrawal of the drug in a 73‐year‐old Japanese woman highlighting the need for clinicians to closely and continuously monitor the development of immune‐related adverse events, including F1DM, irrespective of the number of treatment cycles or the interval after the last immune checkpoint inhibitor cycle.

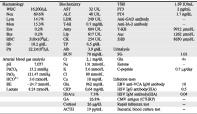

## Introduction

Immune checkpoint inhibitors (ICI) trigger immune‐related adverse events (irAEs) by promoting the activation of cytotoxic T lymphocytes. Binding of the anti‐programmed cell death protein‐1 (PD‐1) antibody to T cells is maintained after a single anti‐PD‐1 antibody injection [1]. Although several cases of irAEs have been reported, to the best of our knowledge, there are no published case reports of fulminant type 1 diabetes mellitus (F1DM) occurring a few months after the cessation of only one or two cycles of atezolizumab administration. Here, we present a case of atezolizumab‐induced F1DM that occurred four months after withdrawal of the drug.

## Case Report

A 73‐year‐old Japanese woman presented with a nodular shadow on a chest X‐ray obtained during a health examination. After surgical lung lobectomy, she was diagnosed with lung adenocarcinoma (T2aN0M0, pathological stage IB) with epidermal growth factor receptor (EGFR) exon 19 deletion and less than 1% of programmed death ligand‐1 (PD‐L1) expression. Two years after the diagnosis of lung cancer, she presented with multiple nodular shadows on chest computed tomography (CT) and was diagnosed with recurrent lung cancer. She received several systemic cytotoxic anti‐cancer agents and EGFR–tyrosine kinase inhibitors after the relapse; however, she showed progressive disease according to the Response Evaluation Criteria in Solid Tumors (RECIST) ver. 1.1. Subsequently, she was treated with two cycles of atezolizumab as sixth‐line chemotherapy, and the treatment was discontinued because of disease progression as per RECIST.

Four months after the cessation of atezolizumab, she developed lower abdominal discomfort followed by dysarthria, gait disorder, fatigue, and vomiting, one day prior to her emergency admission to the hospital. On admission, she had a mild consciousness, with no respiratory failure or any abnormal neurological findings. Although she had Hashimoto's disease and received levothyroxine sodium (50 μg/day), the disease control was satisfactory. Her laboratory findings were serum glucose, 962 mg/dL; blood urea nitrogen, 70 mg/dL; creatinine, 2.10 mg/dL; arterial pH, 7.057; and serum ketone body, high level (Table 1). No evidence of other abnormalities was noted in the peripheral serum examination. Chest and head CT findings showed no signs of worsening in relation to past findings. We considered diabetic ketoacidosis as the cause of reduced consciousness. This condition developed over a rapid period of days, as she had ketoacidosis about a week after the onset of diabetic symptoms, and her serum haemoglobin A1c was slightly elevated. Insulin secretion was depleted despite the absence of pancreatic lesions on imaging, and pancreas‐associated autoantibodies were not detectable. These findings were consistent with the diagnostic criteria for F1DM, and we diagnosed the patient with atezolizumab‐related diabetic ketoacidosis. There were no laboratory findings suggesting new thyroid dysfunction or adrenal insufficiency, which are common endocrine irAEs. Considering that the hypothalamic and pituitary functions were preserved and only the pancreatic endocrine capacity was impaired, we administered continuous intravenous insulin injection. Her serum glucose levels decreased and her symptoms improved. She was prescribed daily insulin injection after discharge.

**Table 1:** Laboratory findings for a female patient with lung adenocarcinoma and atezolizumab‐induced fulminant type 1 diabetes mellitus.

Haematology		Biochemistry		TSH	1.69 IU/mL
WBC	16,200/μL	AST	32 U/L	FT3	2 pg/mL
Neu	69.6%	ALT	48 U/L	FT4	1.7 ng/dL
Lym	14.7%	LDH	249 U/L	Anti‐GAD antibody	−
Mon	15.3%	T‐bil	0.7 mg/dL	Anti‐IA‐2 antibody	−
Eos	0.2%	Amy	604 U/L	T‐KB	9912 μmol/L
Bas	0.2%	Lip	617 U/L	Aac	1262 μmol/L
RBC	310×10^4^/μL	CK	254 U/L	3HB	8650 μmol/L
Hb	10.2 g/dL	TP	6.5 g/dL		
Plt	22.2×10^4^/μL	Alb	3.9 g/dL	Urinalysis	
		BUN	70 mg/dL	SG	1.01
Arterial blood gas analysis		Cr	2.1 mg/dL	Glu	4+
pH	7.057	Na	131 mmol/L	Ketone	−
PaCO_2_	13.2 mmHg	K	5.6 mmol/L	C‐peptide	0.7 μg/day
PaO_2_	121.47 mmHg	Cl	89 mmol/L		
HCO^3−^	3.6 mmol/L	Ca	10 mg/dL	Infection tests	
BE	−24.7 mmol/L	Glu	962 mg/dL	EBV anti‐VCA IgM antibody	10
Lactate	8.24 mmol/L	CRP	0.64 mg/dL	HSV IgG antibody(EIA)	0.5
		HbA1c	7.3%	HSV IgM antibody(EIA)	0.04
		GA	26.8%	CMV antigen (C7HRP)	−
		Cortisol	26 μg/dL	Rapid influenza test	−
		ACTH	19 pg/mL	Bacterial blood culture test	−

3HB, 3‐hydroxybutyrate; AAc, acetoacetate; ACTH, adrenocorticotropic hormone; Alb, albumin; ALT, alanine aminotransferase; Amy, amylase; anti‐GAD antibody, anti‐glutamic acid decarboxylase antibody; anti‐IA‐2 antibody, anti‐insulinoma‐associated antigen‐2 antibody; AST, aspartate transaminase; Bas, basophil; BE, base excess; BUN, blood urea nitrogen; C7HRP, C7 horseradish peroxidase; Ca, calcium; CK, creatine kinase; Cl, chlorine; CMV, cytomegalovirus; Cr, creatinine; CRP, C‐reactive protein; EBV, Epstein–Barr virus; EIA, enzyme immunoassay; Eos, eosinophil; FT3, free triiodothyronine; FT4, free thyroxine; GA, glycoalbumin; Glu, glucose; Hb, haemoglobin; HbA1c, haemoglobin A1c; HCO^3−^, hydrogen carbonate; HSV, herpes simplex virus; Ig, immunoglobulin; K, potassium; LDH, lactate dehydrogenase; Lip, lipase; Lym, lymphocyte; Mon, monocyte; Na, sodium; Neu, neutrophil; PaCO_2_, partial pressure of carbon dioxide; PaO_2_, partial pressure of oxygen; pH, potential of hydrogen; Plt, platelet; RBC, red blood cell count; SG, specific gravity; T‐bil, total bilirubin; T‐KB, total ketone bodies; TP, total protein; TSH, thyroid‐stimulating hormone; VCA, virus capsid antigen; WBC, white blood cell count.

## Discussion

PD‐L1 expressed on pancreatic islet cells connects to PD‐1 on T cells, inhibiting autoimmune responses. When ICIs are administered, this inhibitory mechanism is attenuated by activation of effector T cells against self‐antigens, and they can attack the pancreatic islet cells [2, 3]. Destruction of pancreatic β‐cells by such an autoimmune mechanism may cause absolute insulin deficiency, resulting in type 1 diabetes mellitus (DM). In this case, the patient suddenly developed DM with insulin depletion. No other causes such as bacterial or viral infection, past or family history, or the appearance of autoantibodies were noted. Therefore, the atezolizumab‐associated β‐cell destruction could be a probable cause for the development of DM.

According to a safety study report for ICIs, the incidences of nivolumab‐associated and pembrolizumab‐related DM were 0.33% and 0.14%, respectively. The mean duration between the first ICI initiation date and date of onset of type 1 DM as an irAE was reported to be 155 ± 123 days (range 13–504 days) [4]. The duration was 145 days in this case. Most studies on ICI‐related DM reported DM development during continuous ICI administration; only few studies reported DM development after an interval following the last ICI cycle, as observed in our case.

Binding of the anti‐PD‐1 antibody to T cells is reportedly maintained for more than 20 weeks after a single anti‐PD‐1 antibody injection [1]. This concept was strongly supported in the present case; despite only two cycles of atezolizumab treatment, binding of ICI persisted for a long time.

F1DM often develops because of the destruction of pancreatic β‐cells triggered by several viral infections, caused by enterovirus, herpes simplex virus, rotavirus, and influenza virus, against a background of genetic factors [5]. The present case showed lower abdominal discomfort six days before admission, and there were no laboratory findings suggesting herpes or influenza. We were not able to exclude the possibility that an immune response to viral enteritis triggered the development of F1DM against the background of atezolizumab effect.

In this case, urinary ketones were negative, which we consider was false negative because the test tape used here was acetoacetate dependent. A home urine glucose check may be useful for early diagnosis. In conclusion, we observed a case of F1DM that occurred four months after the cessation of atezolizumab, which was administered in only two cycles. Immunotherapies may lead to serious and unexpected adverse events. Clinicians should closely and continuously monitor the development of irAEs, including F1DM, irrespective of the number of treatment cycles or the interval after the last ICI cycle.

### Disclosure Statement

Appropriate written informed consent was obtained for publication of this case report and accompanying images.
